# The Association between Cesarean Delivery on Maternal Request and Method of Newborn Feeding in China

**DOI:** 10.1371/journal.pone.0037336

**Published:** 2012-05-18

**Authors:** Xinxue Liu, Jun Zhang, Yinghui Liu, Yangmei Li, Zhu Li

**Affiliations:** 1 School of Public Health, Peking University Health Science Center, Beijing, People's Republic of China; 2 MOE and Shanghai Key Laboratory of Children's Environmental Health, Xinhua Hospital, Shanghai Jiao Tong University School of Medicine, Shanghai, People's Republic of China; 3 Department of Obstetrics and Gynecology, Peking University First Hospital, Beijing, People's Republic of China; 4 Department of Public Health and Primary Care, Institute of Public Health, School of Clinical Medicine, University of Cambridge, Cambridge, United Kingdom; Institute of Clinical Effectiveness and Health Policy, Argentina

## Abstract

**Background:**

Cesarean delivery has increased significantly during the last decades. This study aimed to investigate the association between planned mode of delivery and method of feeding.

**Methodology/Principal Findings:**

A cohort was created retrospectively using data from a population-based maternal and child health surveillance system, which covers 27 study sites in China from 1993 to 2006. The cohort consisted of 431,704 women for analysis, including 22,462 women with planned cesarean delivery on maternal request (CDMR) and 409,242 women with planned vaginal delivery (VD). Logistic regression models were used to examine the association between mode of delivery and method of feeding adjusting for selected covariates. In this cohort, 398,176 (92.2%) women exclusively breastfed their baby, 28,798 (6.7%) women chose mixed feeding, and 4,730 (1.1%) women chose formula feeding before hospital discharge. Women who planned CDMR were less likely to exclusively breastfeed and more likely to formula feed their babies than those who planned VD. After adjusting for covariates, the odds ratios were 0.85 (95% CI: 0.81–0.89) for exclusive breastfeeding and 1.61 (95% CI: 1.45–1.79) for formula feeding. Associations between planned mode of delivery and method of feeding in the south, north, rural and urban areas yielded similar results.

**Conclusion:**

This study demonstrated that planned CDMR was associated with a lower rate of exclusive breastfeeding and a higher rate of formula feeding in a low-risk Chinese population.

## Introduction

Cesarean delivery has become an important public health concern worldwide due to its dramatic increase in the last two decades [1], [2]. For example, cesarean delivery rate in the United States increased from 20.7% in 1996 to 30.2% in 2005 [3], while in southeast China it increased from 22% in 1994 to 56% in 2006 [4]. Several studies suggested that the increased use of cesarean delivery on maternal request (CDMR) is one of the main reasons for the steep increase in cesarean delivery rate in China [4]–[6].

Breastfeeding is considered the ideal way to provide nutrition to infants [7], and can significantly reduce infant morbidity [8]. Therefore, the World Health Organization (WHO) recommends exclusive breastfeeding for at least 6 months [9], [10]. It has been well established that women who undergo cesarean delivery are less likely to initiate breastfeeding and exclusively breastfeed their baby compared to those who have a vaginal delivery [11]–[17]. However, this finding should be interpreted with caution as cesarean delivery is often associated with pregnancy complications, which may affect a woman's decision and ability to initiate breastfeeding. In women who do not have such pregnancy complications, does cesarean delivery affect breastfeeding? In our study, we aimed to investigate the association between mode of delivery and method of newborn feeding in a low-risk Chinese population by comparing planned cesarean delivery on maternal request and planned vaginal delivery.

## Methods

The current study is a secondary analysis of data collected by the Perinatal Health Care Surveillance System (PHCSS) from 1993–2006 in 27 study sites in China. The PHCSS was established by the Institute of Reproductive and Child Health (IRCH) at Peking University Health Science Center as an essential component of a population-based China-U.S. Collaborative Project for Neural Tube Defect Prevention [18]. Details of the PHCSS have been described elsewhere [4], [19]. Between 1993 and 2000, every woman in the study sites was given a booklet with a unique identification number. All demographics, medical and reproductive history, prenatal care of the current pregnancy, complications, delivery summary, and maternal and infant health status at each prenatal and postpartum visit were recorded in the booklet by the local health care providers. The booklet was collected and the information was entered into a database after the postpartum period. In 2000, an electronic record system was introduced to some sites to replace the hard copy booklet to facilitate data collection. Data collected in the study sites were sent to the IRCH for data cleaning. After data cleaning, enquiries for missing and erroneous data were sent back to hospitals for ascertainment.

### Study Population

**Figure 1:**
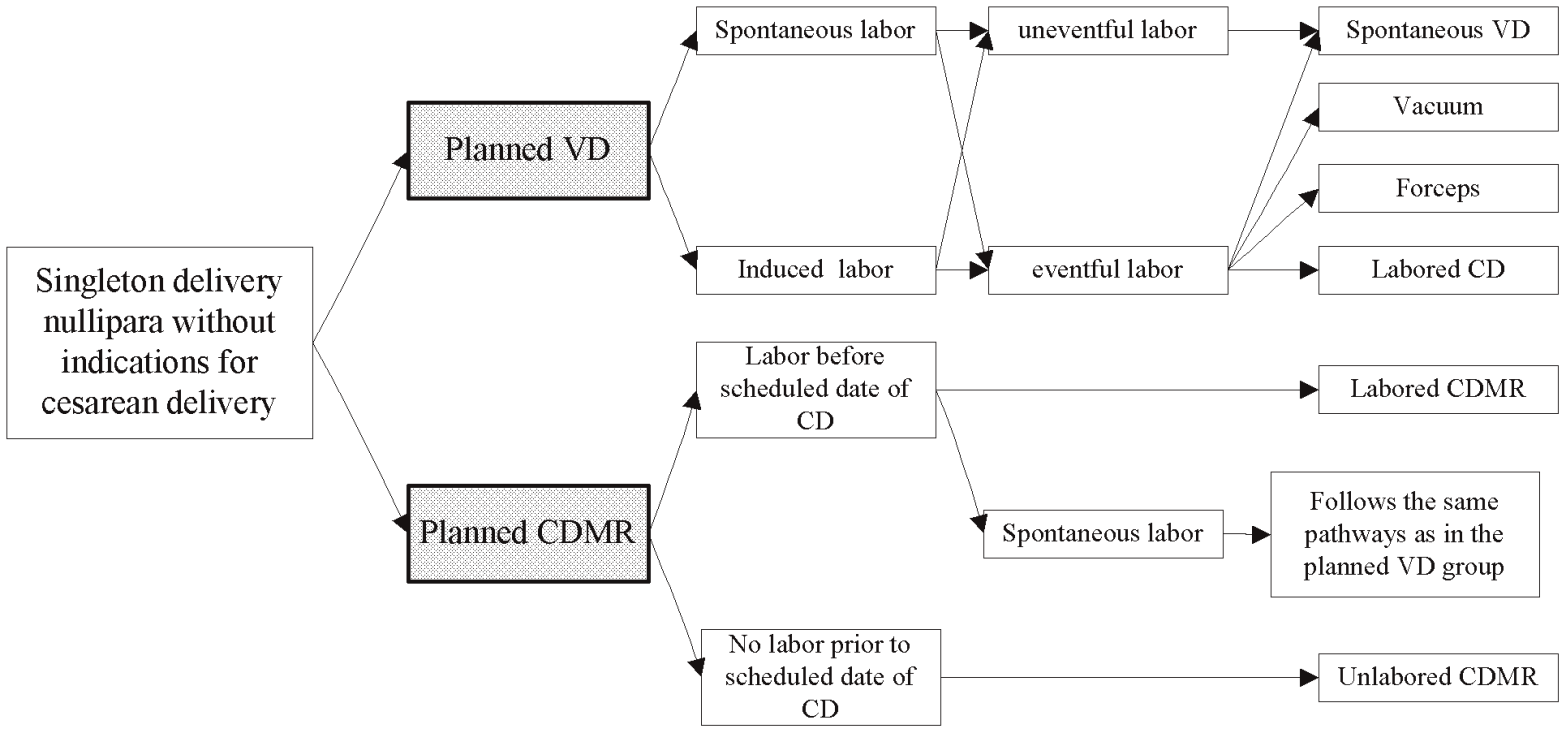
The possible pathways of planned CDMR and planned VD.

From 1993 to June 2006, 1,920,343 women were registered in the PHCSS from the 27 study sites. A cohort was created retrospectively according to the possible pathways of planned CDMR and planned VD as shown in Figure 1. In this retrospective cohort, planned CDMR was defined as the exposure, while women who planned VD were classified as the reference. Planned mode of delivery was inferred from two key delivery variables in the PHCSS: mode of delivery and indications for cesarean delivery. The first variable was listed as spontaneous vaginal, assisted breech, breech extraction, vacuum, forceps, cesarean delivery before the onset of labor, cesarean delivery after the onset of labor, and other, while the second one included fetal distress, cephalopelvic disproportion, breech/transverse presentation, maternal complications, woman's request, previous cesarean section, and other. The indications for cesarean delivery were selected as they were frequently cited in clinical practice in China and the “other” category included rare reasons such as congenital anomalies. Both of mode of delivery and indications for cesarean delivery were collected by midwives after each woman's delivery. Besides the two key variables, several other variables were used to define planned CDMR and planned VD (Table 1).

**Table 1:** Definition of planned CDMR and planned VD (adapted from the paper published by Zhang et al.) [4].

	Planned CDMR	Planned VD
Inclusion criteria	Singleton gestation	Singleton gestation
	Nullipara	Nullipara
	Labored or unlabored cesarean delivery based on “woman's request”	spontaneous vaginal, vacuum, forceps, labored cesarean delivery not based on “woman's” request
	Live fetus prior to delivery	Live fetus prior to delivery
	Gestational age at 38 weeks or later	Gestational age at 38 weeks or later
Exclusion criteria	Malpresentation	Malpresentation
	Placenta previa	Placenta previa
	Antepartum placenta abruption	Antepartum placenta abruption
	Antepartum severe preeclampsia or eclampsia	Antepartum severe preeclampsia or eclampsia
	Abnormal fetal heart rate before labor	Abnormal fetal heart rate before labor
	Suspected fetal growth restriction	Suspected fetal growth restriction
	Suspected large-for-gestational-age fetus	Suspected large-for-gestational-age fetus

**Figure 2:**
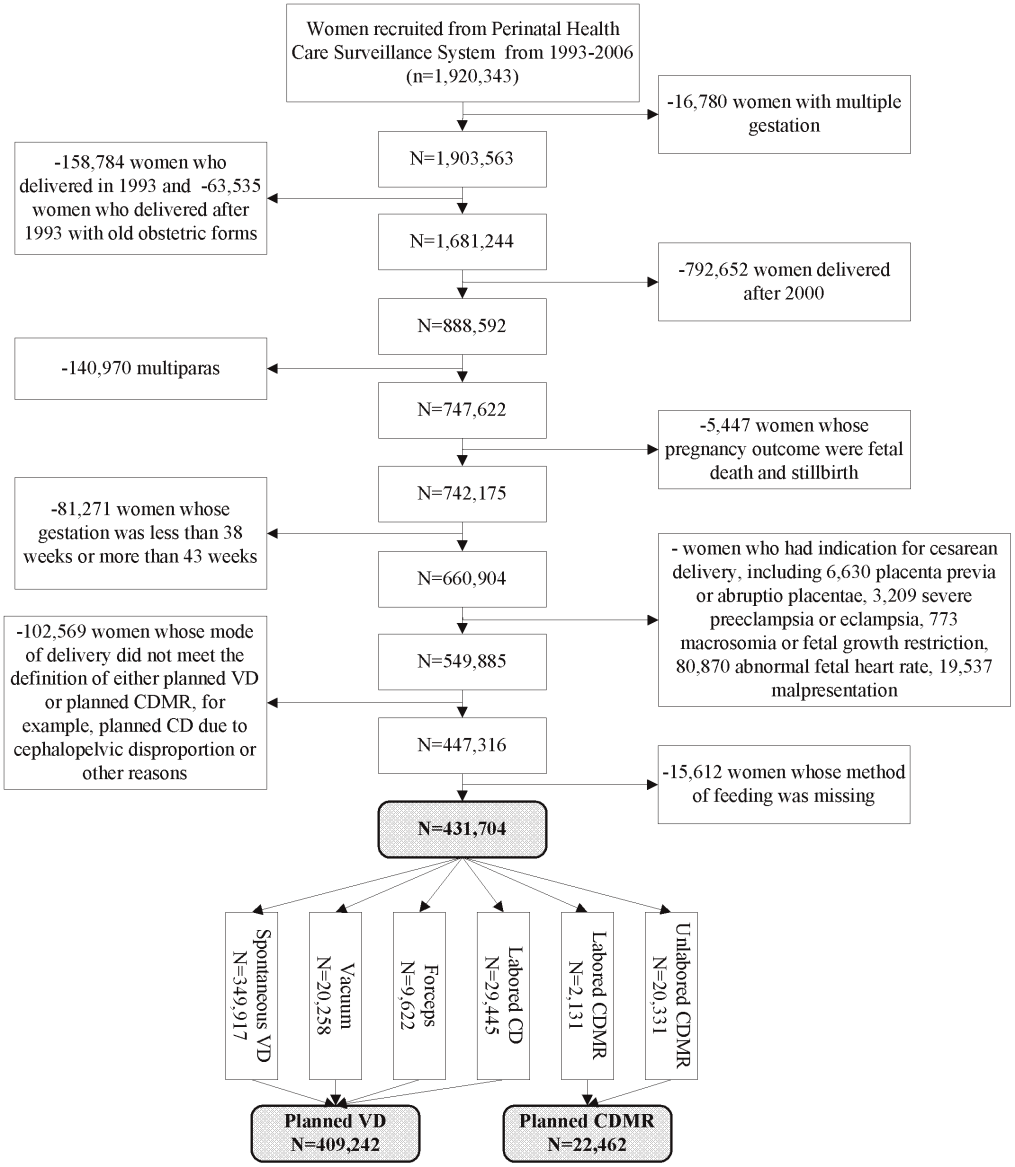
The data processing to create the cohort of planned CDMR and planned VD.

Due to the one-child family policy in China, most families (85.5%) had only one child during the study period. In addition, previous mode of delivery can affect women's delivery choice. Therefore, the study population was restricted to primiparae. From January to September 1993, women in the PHCSS used an old version of the perinatal booklet in which indications for cesarean delivery were not available. Therefore, women who used this perinatal booklet were excluded from the study. Women recruited after 2000 by the electronic record system were also excluded, because the percentage of women with missing information on method of feeding was very high. Further, women with multiple gestations, women whose pregnancy outcomes were either fetal death or stillbirth, women whose babies were either preterm or post-mature, women with indications for cesarean delivery, women who did not satisfy the definition of either planned CDMR or planned VD, and women with missing information on method of feeding were also excluded. Figure 2 shows the details of the sample selection process. Finally there were 431,704 women in the cohort for analysis, including 22,462 women with planned CDMR and 409,242 women with planned VD. Among women with planned CDMR, 2,131 women ended with labored CDMR and 20,331 women ended with unlabored CDMR, while in the planned vaginal delivery group, there were 29,445, 29,880, and 349,917 women who ended up with labored cesarean delivery, assisted vaginal delivery and spontaneous vaginal delivery, respectively

### Study Variables

The main outcome of interest in the current study was method of feeding. During the postpartum examination before hospital discharge, health care providers asked mothers to recall their feeding behavior on that day and recorded the information. There were three categories of feeding: “exclusive breastfeeding”, “mixed feeding”, and “formula feeding”. The definitions of the three categories in this study are as follows:

#### Exclusive breastfeeding

Breastfeeding without any other food, liquid or water;

#### Mixed feeding

Breastfeeding with other food, liquid or water, with breast milk accounting for 20% or more of a baby's total daily food intake;

#### Formula feeding

No Breastfeeding, or breastfeeding with other food, liquid or water, with breast milk accounting for less than 20% of a baby's total daily food intake.

These categories were combined to create two dichotomous outcome variables:

#### Exclusive breastfeeding

Exclusive breastfeeding or not exclusive breastfeeding;

#### Formula feeding

Formula feeding or not formula feeding.

Based on previous literature and data availability in the PHCSS, the following variables were identified as covariates: maternal age at delivery, gestation, maternal body mass index (BMI) at first prenatal visit (<18.5, 18.5–22.9, 23–27.4, and ≥27.5 kg/m^2^), birthweight (<2500, 2500–2999, 3000–3499, 3500–3999, and ≥4000 g), year of delivery (1994–1996, 1997–1998, and 1999–2000), site of delivery (north/south), location of residence (urban/rural), occupation (farmer, local enterprise worker, worker, professional, business, and other), educational level (attended primary school or less, middle school, high school, and college or above), delivery hospital level (city/provincial, county, township, and other), infant sex (male/female), and mother's hospital stay before discharge (≤3, 4–7, and >7days). BMI and birthweight were recoded into categorical variables in the analysis for two reasons. First, the power of the analysis was increased by recoding the missing values of these variables as one category rather than excluding them from the analysis. Second, it was suspected that the association between these variables and breastfeeding may not be linear. Sensitivity analysis was carried out using continuous variables. The results remained the same.

### Statistical Analysis

Analysis of variance (ANOVA) and chi-square tests were used to explore the differences in demographic variables between the planned VD and planned CDMR groups, for continuous and categorical variables respectively. Means and standard deviations (SD) for continuous variables, as well as numbers (N) and percentages (%) for categorical variables were presented. Logistic regression models were then used to examine the associations between planned mode of delivery and exclusive breastfeeding, and between planned mode of delivery and formula feeding. These were then repeated with covariates included in the models. Crude odds ratios (OR) and odds ratios adjusted for all covariates were reported in this study. Sensitivity analysis was carried out to explore the associations between planned mode of delivery and method of feeding stratified by site of delivery and location of residence. The results for actual route of delivery were also reported to explore the mechanism for the relationship between delivery and breastfeeding. All analyses were performed using SPSS version 11.5 (SPSS Inc.). All *P* values presented are two tailed, and a *P* value of <0.05 was considered statistically significant. Written informed consent was received from all participants in the PHCSS. The current study using de-identified registry data was approved by the ethics committee at the Peking University Health Science Centre.

## Results

Overall, there were 22,462 (5.2%) women who planned CDMR. Table 2 shows the maternal and infant demographics according to planned mode of delivery. In the present cohort, 398,176 (92.2%) women exclusively breastfed their baby, 28,798 (6.7%) women chose mixed feeding, and 4,730 (1.1%) women chose formula feeding before hospital discharge. Women with planned CDMR had a higher formula feeding rate (2.1%) and a lower exclusive breastfeeding rate (89.7%) than women with planned VD (1.0% and 92.4% respectively). All maternal and infant demographics were found to be significantly different between the two groups (Table 2).

**Table 2:** Description of maternal and infant characteristics by planned mode of delivery^a^.

		Planned VD	Planned CDMR	*P*
**N (%)**		409,242 (94.8)	22,462 (5.2)	
**Maternal age at delivery (yrs)**		24.2 (2.4)	25.2 (2.6)	<0.01
**BMI (kg/m^2^)**		20.5 (2.2)	20.7 (2.5)	<0.01
**Gestation (weeks)**		40.1 (1.0)	40.2 (1.0)	<0.01
**Birthweight (g)**		3,332 (375)	3,438 (393)	<0.01
**Hospital stay (days)**		5.0 (3.1)	7.6 (2.5)	<0.01
**Year of delivery**	1994–1996	193,650 (47.3)	3,531(15.7)	<0.01
	1997–1998	117,624 (28.7)	5,993 (26.7)	
	1999–2000	97,968 (23.9)	12,938 (57.6)	
**Site of delivery**	North	84,159 (20.6)	3,160 (14.1)	<0.01
	South	325,083 (79.4)	19,302 (85.9)	
**Location of residence**	Urban	84,441 (20.6)	6,749 (30.0)	<0.01
	Rural	324,801 (79.4)	15,713 (70.0)	
**Occupation**	Farmer	229,427 (56.1)	9,806 (42.7)	<0.01
	Rural enterprise worker	72,755 (17.8)	3,195 (14.2)	
	Worker	59,099 (14.4)	4,082 (18.2)	
	Professional	24,305 (5.9)	2,699 (12.0)	
	Business	6,762 (1.7)	674 (3.0)	
	Other	14,383 (3.5)	1,851 (8.2)	
**Education**	College or above	20,250 (4.9)	2,360 (10.5)	<0.01
	High school	64,728 (15.8)	5,736 (25.5)	
	Middle school	266,666 (65.2)	12,539 (55.8)	
	Primary school or less	56,508 (13.8)	1,754 (7.8)	
**Hospital level**	City or Provincial	75,730 (18.5)	6,680 (29.7)	<0.01
	County	140,798 (34.4)	11,603 (51.7)	
	Township	184,336 (45.0)	4,096 (18.2)	
	Other	7,440 (1.8)	33 (0.1)	
**Infant sex**	Male	207,305 (50.7)	11,713 (52.1)	<0.01
	Female	201,493 (49.2)	10,717 (47.7)	
**Method of feeding**	Exclusive breastfeeding	378,031 (92.4)	20,145 (89.7)	<0.01
	Mixed feeding	26,961 (6.6)	1,837 (8.2)	
	Formula feeding	4,250 (1.0)	480 (2.1)	

a *Data shown are mean (SD) for continuous variables and number (percentage) for categorical variables; Percentages for each categorical variable do not always add up to 100% because of missing data; CDMR: cesarean delivery on maternal request; VD: vaginal delivery.*

In the analysis for the outcome of exclusive breastfeeding, the crude OR for planned CDMR compared to planned VD was 0.72 (95% CI: 0.69–0.75) (Table 3). After adjusting for maternal age at delivery, BMI, gestation, infant birthweight, delivery year, site of delivery, location of residence, occupation, educational level, delivery hospital level, infant sex, and mother's hospital stay before discharge, the OR became 0.85 (95% CI: 0.81–0.89). In subgroup analyses, the ORs for planned CDMR compared to planned VD in the adjusted model were 0.87 (95% CI: 0.77–0.98) in the north and 0.84 (95% CI: 0.80–0.89) in the south. Compared to women who planned VD, those who planned CDMR were 7% (95% CI: 0.85–0.98) less likely to exclusively breastfeed their babies in the urban area and 20% (95% CI: 0.75–0.86) less likely in the rural area (Table 3).

**Table 3:** Logistic regression models for the association between planned mode of delivery and method of feeding^a^.

	Exclusive breastfeeding^b^			Formula feeding^c^		
	N (%)	Crude OR (95% CI)	Adjusted OR (95% CI)^d^	N (%)	Crude OR (95% CI)	Adjusted OR (95% CI)^d^
Total	398,176 (92.2)	0.72 (0.69–0.75)	0.85 (0.81–0.89)	4,730 (1.1)	2.08 (1.89–2.29)	1.61 (1.45–1.79)
**Stratified by site of delivery**						
North	82,219 (94.2)	0.44 (0.39–0.49)	0.87 (0.77–0.98)	796 (0.9)	4.20 (3.41–5.17)	2.02 (1.61–2.54)
South	315,957 (91.7)	0.80 (0.76–0.84)	0.84 (0.80–0.89)	3,934 (1.1)	1.79 (1.60–1.99)	1.52 (1.35–1.71)
**Stratified by location of residence**						
Urban	75,636 (82.9)	0.84 (0.79–0.90)	0.93 (0.85–0.98)	1,973 (2.2)	1.93 (1.69–2.20)	1.63 (1.41–1.89)
Rural	322,540 (94.7)	0.80 (0.75–0.86)	0.80 (0.75–0.86)	2,757 (0.8)	1.81 (1.58–2.08)	1.55 (1.33–1.80)

a *Data shown are odds ratio (95% confidence interval) and number (percentage);*

b *Odds ratio expresses the likelihood that mothers will exclusively breastfeed their babies before discharge in the planned CDMR group compared to that in the planned VD group;*

c *Odds ratio expresses the likelihood that mothers will formula feed their babies before discharge in the planned CDMR group compared to that in the planned VD group;*

d *Adjusted for maternal age at delivery, BMI, gestation, infant birthweight, year of delivery, occupation, educational level, delivery hospital level, infant sex, mother's hospital stay before discharge, and location of residence or site of delivery or both.*

For the outcome variable of formula feeding, the crude OR and adjusted OR for planned CDMR compared to planned VD were 2.08 (95% CI: 1.89–2.29) and 1.61 (95% CI: 1.45–1.79) (Table 3). In the north, the ORs in the crude and adjusted models were 4.20 (95% CI: 3.41–5.17) and 2.02 (95% CI: 1.61–2.54) respectively, and in the south 1.79 (95% CI: 1.60–1.99) and 1.52 (95% CI: 1.35–1.71) respectively. Women who planned CDMR were 63% (95% CI: 1.41–1.89) more likely to formula feed their babies than those who planned VD in the urban area and 55% (95% CI: 1.33–1.80) in the rural area (Table 3).

Table 4 shows the association between actual route of delivery and method of breastfeeding. Compared to spontaneous vaginal delivery, the ORs and 95% CIs of exclusive breastfeeding in the fully adjusted model for unlabored CDMR, labored CDMR, labored cesarean delivery and assisted vaginal delivery were 0.81 (0.77–0.85), 0.92 (0.79–1.07), 0.90 (0.86–0.94), and 0.85 (0.82–0.89), respectively, and the ORs and 95% CIs of formula feeding were 1.79 (1.59–1.99), 1.38 (0.98–1.94), 1.27 (1.13–1.43), and 1.35 (1.21–1.50), respectively.

**Table 4:** Logistic regression models for the association between actual route of delivery and method of breastfeeding^a^.

Planned mode of delivery	Actual route of delivery	Exclusive breastfeeding^b^			Formula feeding^c^		
		N (%)	Crude OR (95% CI)	Adjusted OR (95% CI)^d^	N (%)	Crude OR (95% CI)	Adjusted OR (95% CI)^d^
Planned VD	Spontaneous VD	324,241 (92.7)	Reference	Reference	3,434 (1.0)	Reference	Reference
	Assisted VD	27,108 (90.7)	0.77 (0.74–0.81)	0.85 (0.82–0.89)	405 (1.4)	1.39 (1.25–1.54)	1.35 (1.21–1.50)
	Labored CD	26,682 (90.6)	0.77 (0.73–0.80)	0.90 (0.86–0.94)	411 (1.4)	1.43 (1.29–1.58)	1.27 (1.13–1.43)
Planned CDMR	Labored CDMR	1,934 (90.8)	0.78 (0.67–0.90)	0.92 (0.79–1.07)	36 (1.7)	1.73 (1.25–2.41)	1.38 (0.98–1.94)
	Unlabored CDMR	18,211 (89.6)	0.68 (0.65–0.71)	0.81 (0.77–0.85)	444 (2.2)	2.25 (2.04–2.49)	1.79 (1.59–1.99)

a *VD: vaginal delivery; CDMR: cesarean delivery on maternal request; CD: cesarean delivery;*

b *Odds ratio expresses the likelihood that mothers will exclusively breastfeed their babies before discharge compared to that in the reference group;*

c *Odds ratio expresses the likelihood that mothers will formula feed their babies before discharge compared to that in the reference group;*

d *Adjusted for maternal age at delivery, BMI, gestation, infant birthweight, year of delivery, location of residence, site of delivery, occupation, education level, delivery hospital level, infant sex, and mother's hospital stay before discharge.*

## Discussion

In this retrospective cohort study, it was found that women with planned CDMR were less likely to breastfeed and more likely to formula feed their babies before hospital discharge compared to those with planned VD. After adjusting for covariates, the odds ratios were 0.85 (95% CI: 0.81–0.89) for exclusive breastfeeding and 1.61 (95% CI: 1.45–1.79) for formula feeding.

The exclusive breastfeeding rate before hospital discharge was 92.2% in the current study, which is similar to previous studies in China [20]. In 2009, Xu et al. systematically reviewed breastfeeding in China and reported that the exclusive breastfeeding rate at hospital discharge ranged from 76.1% to 97.3% across the country [20]. In that review, the study periods of the studies included in the review ranged from 1994 to 2004, which is consistent with the current study.

It is believed that there are two biological mechanisms of the association between mode of delivery and breastfeeding. The first is associated with surgery, for example, long duration of separation of mother and the newborn due to complications of the surgery, such as pain, hemorrhage, and infections [21]. The other biological mechanism is related to labor via a hormone called prolactin. Prolactin plays an important role in the process of lactogenesis [22]–[24]. Wang et al. found that the postpartum serum prolactin level was lower in the elective unlabored cesarean delivery group than in the vaginal delivery group. These two biological mechanisms could probably explain the association between actual route of delivery and rate of breastfeeding in the current study in which unlabored CDMR women had the highest risk of formula feeding before hospital discharge, followed by labored CDMR, labored cesarean delivery, and assisted vaginal delivery. Women who underwent spontaneous vaginal delivery were at the lowest risk (Table 4). Results from several previous studies also support the two mechanisms [25], [26]. For example, Zanardo et al. reported that unlabored cesarean delivery was associated with a decreased rate of breastfeeding initiation at discharge compared to emergency labored cesarean delivery and vaginal delivery [26]. Liston et al. found that women with unlabored cesarean delivery were 26% less likely to breastfeed their babies at discharge than those with assisted vaginal delivery, while women with labored cesarean delivery were only 7% less likely to breastfeed at discharge [25].

It is possible that the association between planned mode of delivery and breastfeeding can simply be explained by the correlation of maternal intentions to delivery and feeding, which means women who choose vaginal delivery are simply more likely to choose breastfeeding compared to those who planned cesarean delivery. However, this is not the case in our study. Women who chose the same delivery method can have significantly different breastfeeding rate. For example, among women who planned to deliver vaginally, those ended with cesarean delivery are 10% less likely to breastfeed their babies than those ended with vaginal delivery (Table 4). On the other hand, women who planned different delivery methods can have a similar breastfeeding rate in our study (Table 4).

Several studies have found that cesarean delivery is a barrier for breastfeeding [11]–[17], which is consistent with the current study results in a low-risk Chinese population. However, all previous studies used actual route of delivery as the measurement of exposure, while in the current study we analyzed the effects of delivery on breastfeeding by planned mode of delivery. Although the biological mechanism underlying the association between planned mode of delivery and method of feeding is through actual route of delivery, the association with planned mode of delivery provides more concrete evidence to inform the decision making of women or health care practitioners during pregnancy regarding mode of delivery [1], since it highlights the risks of possible ending delivery modes at the time of decision making.

The increasing trend of CDMR was also observed in other countries [27], [28]. However, the impacts of CDMR on children and mothers' health are still largely unknown. In the current study, we found that the CDMR can impede breastfeeding. This information should be taken into account in clinical practice when counseling women about the mode of delivery, especially those without medical indications for cesarean delivery.

The present study has several advantages. First of all, this retrospective cohort study was based on prospectively collected data in the population-based PHCSS with large sample size and comprehensive coverage in the study sites. Potential confounders were well documented and comprehensively adjusted in the analysis. Secondly, the selection criteria for the cohort were strict to exclude the effects of potential biological confounders, which are hard to measure and control. In addition, the mode of delivery was identified based on the delivery records in the PHCSS and thus was more accurate than that collected by questionnaire in some of the previous studies. Finally, maternal request was listed as one of the indications for cesarean delivery, providing a unique chance to study the effects of CDMR in a low-risk population. Nonetheless, the study has several limitations. First, indications for cesarean delivery were recorded by midwives, and have not been independently verified [4]. However, the cesarean delivery rate for breech presentation remained unchanged over the study period and was well within the normal range, indicating the reliability of the data [4]. Second, since the study population was restricted to singleton primiparous women, the findings may be not applicable to multiparous women or women with multiple births. In addition, since the cohort was retrospectively created according to the actual route of delivery, women who planned a cesarean delivery but labored and had a vaginal delivery cannot be distinguished from those who planned and had a vaginal delivery. These women were therefore included in the planned VD group. However, the number of these misclassified women is relatively small compared to the whole cohort and thus the misclassification is unlikely to affect the results. Finally, detailed information about breastfeeding after hospital discharge was not available in the PHCSS, thus the association between planned mode of delivery and long-term feeding methods could not be analyzed. However, the method of feeding before hospital discharge is very important and can affect breastfeeding durations in later life [26].

In conclusion, planned CDMR was associated with a lower rate of breastfeeding. This information is important when physicians are counseling women who are considering CDMR. In addition, clinical practitioners should provide more prenatal and postpartum support for women planning CDMR to initiate and continue breastfeeding than for those considering vaginal delivery. Future studies on the long-term impacts of CDMR on children and mothers' health are needed.
